# CD36 and Its Role in Regulating the Tumor Microenvironment

**DOI:** 10.3390/curroncol29110642

**Published:** 2022-10-27

**Authors:** Xinzhi Liao, Sheng Yan, Jialin Li, Chengming Jiang, Sigen Huang, Shengyin Liu, Xiaofeng Zou, Guoxi Zhang, Junrong Zou, Quanliang Liu

**Affiliations:** 1 The First Clinical College, Gannan Medical University, Ganzhou 341000, China; 2 Department of Urology, The First Affiliated Hospital of Gannan Medical University, Ganzhou 341000, China

**Keywords:** CD36, lipid metabolism, tumor-associated immune cells, angiogenesis

## Abstract

CD36 is a transmembrane glycoprotein that binds to a wide range of ligands, including fatty acids (FAs), cholesterol, thrombospondin-1 (TSP-1) and thrombospondin-2 (TSP-2), and plays an important role in lipid metabolism, immune response, and angiogenesis. Recent studies have highlighted the role of CD36 in mediating lipid uptake by tumor-associated immune cells and in promoting tumor cell progression. In cancer-associated fibroblasts (CAFs), CD36 regulates lipid uptake and matrix protein production to promote tumor proliferation. In addition, CD36 can promote tumor cell adhesion to the extracellular matrix (ECM) and induce epithelial mesenchymal transition (EMT). In terms of tumor angiogenesis, CD36 binding to TSP-1 and TSP-2 can both inhibit tumor angiogenesis and promote tumor migration and invasion. CD36 can promote tumor angiogenesis through vascular mimicry (VM). Overall, we found that CD36 exhibits diverse functions in tumors. Here, we summarize the recent research findings highlighting the novel roles of CD36 in the context of tumors.

## 1. Introduction

The tumor microenvironment (TME) includes proliferating tumor cells, fibroblasts, immune-related cells, tumor microvessels, cytokines and the extracellular matrix [1]. Many studies have shown that TME can normalize tumor cells, suggesting that the modification of TME may be an effective therapeutic strategy for cancer [2]. Dyshomeostasis of lipid metabolism is one of the most prevalent metabolic transformations in TME [3,4]. Through the reprogramming of the lipid metabolism, cancer cells can obtain a large amount of energy, biofilm components, and biological signal molecules from the surrounding environment to promote proliferation, invasion, and metastasis of cancer cells [5]. Therefore, it is vital to alter lipid metabolism in cancer to limit its progression.

CD36, a key receptor for lipid transport, plays an essential role in the regulation of lipid metabolism in tumors. For example, omental adipocytes promote the migration and invasion of OvCa cells by upregulating CD36 expression in OvCa cells and thereby promoting their lipid accumulation. Inhibition of CD36 prevents the development of this malignant phenotype [6]. Promoting oral cancer or melanoma metastasis in mice, by using palmitic acid diet, involves the mediation of CD36 [7]. CD36 also plays an important role in regulating tumor angiogenesis. Binding of TSP-1 to CD36 on microvascular endothelium inhibits angiogenesis [8], but CD36 could induce VM formation in melanoma cells by promoting the adhesion of tumor cells [9]. Additionally, high expression of CD36 in tumor cells was also found to predict poor prognosis [10,11]. CD36 also plays an important role in tumor metastasis [12], affecting tumor development through different mechanisms.

## 2. Structure and Distribution of CD36

**Figure 1:**
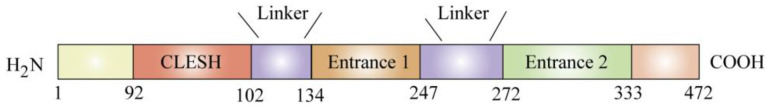
CD36 primary structure. The CLESH structural domain in CD36 can regulate tumor angiogenesis by interacting with the TSR. CD36 forms two hydrophobic lumens (entrance 1 and entrance 2) in its pericyte region and binds to hydrophobic lipid-associated proteins to regulate cellular lipid metabolism.

CD36 is a secondary transmembrane glycoprotein with a molecular weight of approximately 88 kDa and is part of the class B receptor family (SR-B2), which is known as a fatty acid transporter (FAT); it is also referred to as platelet GPIV and GP88 [13,14]. The protein has an extracellular region containing a ligand binding site, two transmembrane fragments, and a short cellular cytoplasmic tail at both ends (N and C) [15]. CD36 forms two hydrophobic lumens in its pericyte region and binds to hydrophobic lipid-associated proteins, such as long chain fatty acids (LCFA), oxidized low density lipoprotein (oxLDL), and oxidized phospholipids (oxPLs), to regulate cellular lipid metabolism [16]. In addition, the CD36, LIMP-2, Emp sequence homologous (CLESH) structural domain can regulate tumor angiogenesis by interacting with the TSP-1 repeat domain 2 (TSR) [17] (Figure 1).

CD36 has been shown to be expressed in various cell types such as adipocytes, platelets, mononuclear macrophages, microvascular endothelial cells, myocardial cells, dendritic cells, hepatocytes, and tumor cells [18]. The expression level of CD36 is various in different cell types, and it performs diverse functions. CD36 protein has several post-translational modification sites, such as glycosylation, palmitoylation, ubiquitination, phosphorylation and acetylation, which are involved in the regulation of CD36 signaling function [19]. In order to elucidate the role of CD36 in cancer, a comprehensive study of the structure and function of CD36 is necessary.

## 3. CD36 and Tumor-Associated Immune Cells

**Figure 2:**
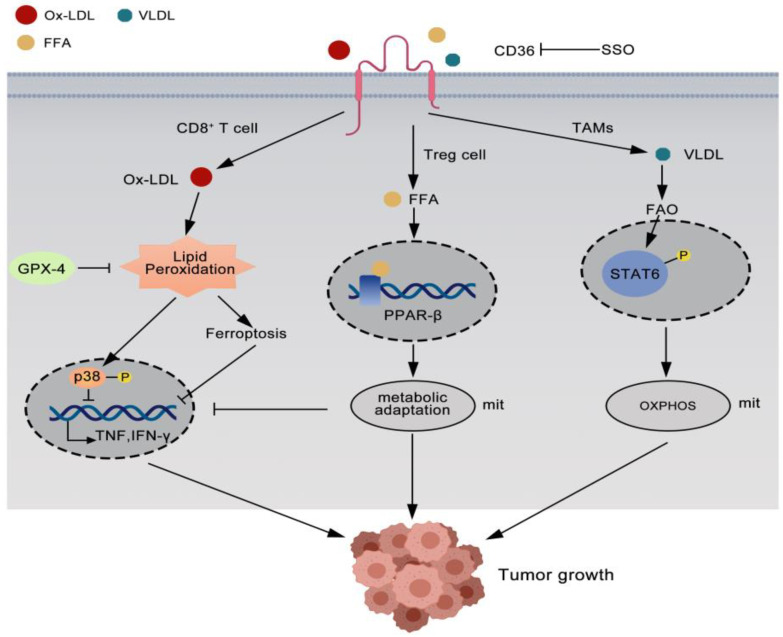
CD36 plays a role in tumor immunity. CD36 mediates Ox-LDL uptake, promotes lipid peroxidation in CD8^+^T cells, phosphorylates p38 kinase, which in turn inhibits IFN-γ and TNF production. CD36 also activates PPAR-β signaling to enhance mitochondrial fitness of Treg cells in tumors. And CD36 promotes STAT6 phosphorylation to enhance mitochondrial fitness of TAMs, which in turn promotes tumor proliferation.

Tumor-associated immune cells play a key role in promoting tumor development in the TME and CD36 also plays an important role in regulating immunity [20,21]. Recent studies have found that CD36-mediated lipid metabolism on tumor-associated immune cells promotes tumor progression (Figure 2).

### 3.1. CD36 Reprogrammed Regulatory Treg Cells

Intratumor Treg cells are a type of T cells produced in the TME. These cells have an immunosuppressive effect and represent a main obstacle to immunotherapy for cancer [22,23]. Under physiological conditions, Treg cells closely monitor and suppress the function of peripheral auto-reactive T and B cells to prevent the progress of autoimmune diseases. In contrast, in immunosuppressive diseases (such as chronic infection and tumor), there is a significant increase in the number of Treg cells, which is a key factor contributing to the failure of anti-infection immunity and anti-tumor immunity. Although the strategy of consuming Treg cells was found to increase the anti-tumor response [24,25], targeting of Treg cells is limited by the severe autoimmunity caused by the systemic reduction in Treg cells and the unnecessary exhaustion of the resultant effector T cells.

CD36 promotes the functional adaptation of intratumor Treg cells. In vivo research has shown increased CD36 expression in intratumoral Treg cells compared to Treg cells in the spleen and draining lymph nodes. This increased expression may promote the survival and functional adaptation of intratumoral Treg cells by supplying lipid signaling to activate the peroxisome proliferator-activated receptor β (PPAR-β) pathway that supports mitochondrial adaptation. Moreover, immunotherapy administered in combination with anti-PD-1mAb further dampened tumor development and extended the survival of TregCD36^−/−^ mice compared to wild-type mice [26]. In addition, in normal tissues, CD36 can also regulate the functional activity of Treg cells [27]. For example, it mediates the transfer of cell surface antigens, supports further bridging of the thymic regulatory T cell receptor (TCR) library in cells, and coordinates the immune maturation of Treg cells [28]. In addition, CD14^+^CD36^hi^ monocytes can provide TGF-β and retinoic acid to induce differentiation and maturation of Foxp^3+^ Treg cells [29]. Treg cells derive energy primarily through β-oxidation of FAs in the periphery. CD36 can activate the PPAR-γ pathway to upregulate fatty acid oxidation (FAO), which in turn enhances UDP-GlcNAc biosynthesis, promoting N-linked glycosylation of TβRII/IL-2Rα and downstream signaling, thereby promoting Foxp3 transcription and increasing Treg cell responsiveness [30]. In vivo mouse experiments indicated that CD36 deficiency was shown to impair the influence of diet on Treg cells [31].

In summary, CD36 has an important synergistic effect on Treg cells by reprogramming the lipid metabolism of Treg cells to adapt to the TME and promote tumor development. Selective targeting of CD36 on Treg cells may achieve better inhibition of tumor progression and better protection of immune homeostasis than the elimination of Treg cells.

### 3.2. CD36 Impairs Antitumor Immunity of CD8^+^T Cells

Tumor-infiltrating CD8^+^T cells are the mainstay of tumor cell killing [32,33], and these cells specifically kill tumor cells mainly through the perforin-granzyme, Fas-FasL, and TNF-TNFR pathways [34]. However, their activity is often inhibited in the TME [35]. Cholesterol was previously reported to induce CD8^+^T cell failure [36] and the modulation of cholesterol metabolism was found to enhance the antitumor response of CD8^+^T cells [37]. This indicates that excessive lipid metabolism affects the antitumor immunity of CD8^+^T cells.

Excess lipids can cause a state of cellular lipid peroxidation through oxidative stress and the induction of reactive oxygen species (ROS) [38]. Ferroptosis is an iron-dependent, iron-induced mode of cell death induced by catalyzing lipid peroxidation of highly expressed unsaturated FAs on cell membranes in the presence of divalent iron or ester oxygenases [39]. In tumor tissue, CD36 can impair the immune surveillance of CD8^+^T cells by promoting the uptake of large amounts of arachidonic acid and inducing iron toxicity, lipid peroxidation, and the reduced production of cytotoxic cytokines by CD8^+^T cells. In addition, the higher the expression of CD36 on CD8^+^T cells, the worse the prognosis of the patient. Furthermore, in mice subjected to genetic ablation, the combination of CD36^−/−^ CD8^+^T cells and anti-PD-1 antibody achieved better anti-tumor effect and prolonged mouse survival than the combination of WT CD8^+^T cells and anti-PD-1 antibody or CD36^−/−^ CD8^+^T cells [40]. This result is similar to that of Wang et al. [26]. GPX4 prevents cellular ferroptosis by eliminating intracellular lipid peroxidation, whereas the inhibition of GPX4 triggers ferroptosis [41]. CD36 inhibits the production of IFN-γ and TNF by mediating OxLDL uptake, causing lipid peroxidation in CD8^+^T cells and downstream P38 kinase activation, thereby weakening the antitumor immunity of CD8^+^T cells [42]. Myeloid-derived suppressor cells (MDSCs) play a role in the pathological processes of tumor immune escape, immune tolerance, and immunosuppression, and these cells have the ability to significantly suppress the antitumor cellular immune response [43]. MDSCs can restrain the antitumor capacity of CD8^+^T cells by promoting their own oxidative metabolism and immunosuppressive function through improved absorption of FAs by CD36 [44].

These findings suggest that CD36 restricts the immune activity of CD8^+^T cells by means of lipid metabolic reprogramming and promotes tumor proliferation and progression.

### 3.3. CD36 Cooperates with Tumor-Associated Macrophages to Promote Tumor Growth

Macrophages that infiltrate tumor tissue or reside in the solid TME are defined as tumor-associated macrophages (TAMs). As an important component of the TME, TAMs regulate numerous key processes in tumors, including stimulating tumor proliferation, induction of angiogenesis, immunosuppression, metastasis, and chemoresistance [45].

Macrophage polarization toward the pro-tumor phenotype depends on intracellular lipid accumulation, which provides a stable energy resource to cater to the high oxygen consumption rate (OCR) and increased oxidative phosphorylation (OXPHOS) of TAMs mitochondria. CD36-mediated uptake of LDL and VLDL supports macrophage polarization toward the M2-like phenotype [46]. Similarly, enhanced polarization of TAMs by PPAR-γ is dependent on CD36-mediated lipid uptake [47]. TAMs were shown to express high levels of CD36 to enable uptake of excess lipids and to use FAO rather than glycolysis to obtain energy. Mitochondrial OXPHOS in TAMs is driven by high levels of FAO, which in turn generates ROS, phosphorylation of JAK1, and dephosphorylation of SHP1, leading to activation of STAT6, which in turn regulates gene transcription for TAMs production and function [48]. CD36 also mediates the transport of non-coding RNAs to increase the recruitment of TAMs in tumors. For example, CD36 was shown to promote the transfer of apoptotic tumor cell-derived miR-375 into TAMs via the lipoprotein pathway, thereby inhibiting the mRNA and protein expression of TNS3 and PXN to promote the infiltration and migration of TAMs; miR-375 was shown to promote TAMs recruitment in tumor tissues by regulating CCL2 expression [49]. In addition, CD36 promotes the integration of TAMs with tumor stem cells to make up extremely aggressive tumor-hybrid cells (THCs). Recognition of cell membrane lipids by CD36 promotes the fusion activity of macrophages [50]. Studies have also found that the lipid uptake function of CD36 promotes the oxidative state and polarization of macrophages, and that highly oxidized macrophages have a high affinity for fusion. Thus, CD36 can promote the fusion of macrophages with tumor stem cells to form THCs, making the tumor highly aggressive [51].

To summarize, CD36 can synergize the pro-cancer effects of TAMs via multiple mechanisms, suggesting that targeting CD36 to modulate the oxidation and polarization of TAMs may be an effective therapeutic strategy.

## 4. CD36 and CAFs

CAFs in tumor development are gradually being discovered [52]. CAFs have an important influence on tumor fibrosis, angiogenesis, migration, and chemotherapy by releasing cytokines, chemokines and collagen into the TME and by mediating collagen crosslinking to increase stromal stiffness and stimulate tumor cell invasion [53,54]. For example, CAFs were shown to secrete miR-92a-3p factor to promote metastasis and induce chemoresistance in colorectal cancer [55]. However, the role of CD36 in CAFs may vary in different tumors or different microenvironments.

CD36 tends to be under-expressed in CAFs [56]. CD36 expression in the cell membrane of fibroblasts was shown to be higher than that in normal tissues, but decreased gradually during transformation of fibroblasts into CAFs, and this process was mainly completed within 24 h [57]. Moreover, the accumulation of matrix proteins in CAFs is also dependent on the low expression of CD36. For example, in breast cancer tissues with high mammographic density, the lower adipocyte content and higher stromal cell and extracellular matrix content were associated with the absence of CD36 expression in CAFs, and the lower CD36 expression was associated with greater tumor aggressiveness [58,59]. In addition, fibroblasts with low CD36 expression produced more collagen and fibronectin than fibroblasts with high CD36 expression [56]. Moreover, Jabbari et al. co-cultured CD36 overexpressing fibroblasts with breast cancer cell lines and found that SLIT3, FBLN1, and PENK secreted by CD36 + fibroblasts can repress the proliferation of breast cancer cells through the apoptotic pathway [60]. This study suggested a negative regulatory role of CD36 in CAFs, and this negative regulatory role may be manifested mainly in breast cancers, especially those with a high mammography density.

However, CD36 expression in CAFs was also found to enhance tumor progression. For example, in colorectal cancer (CRC), CAFs undergo reprogramming of lipid metabolism, mainly in the form of increased FAs, phospholipids, and triglycerides. The increased uptake of lipid metabolites secreted by CAFs mainly through CD36 expressed on CRC cell membrane enhances the migration of tumor cells [61]. In addition, pancreatic cancer cell-derived TGF-β1 was shown to stimulate the recruitment and activation of CAFs and upregulate TSP-2 expression via the p-Smad2/3 pathway. The high expression of TSP-2 in turn promoted the growth and adhesion of pancreatic ductal adenocarcinoma cells by activating the integrin αvβ3/CD36-MAPK pathway [62]. This finding also suggests that CD36 can act in concert with CAFs to increase tumor progression.

In summary, CD36 regulates lipid uptake and stromal protein production between CAFs and TME and regulates tumor progression.

## 5. CD36 Activates EMT

EMT is strongly associated with cancer proliferation and migration [63,64]. CD36 is expressed strongly in the TME and also has a significant function in EMT. For example, FAs released from adipocytes are transported into breast cancer cells via CD36, which in turn induces EMT and stem cell properties in breast cancer cells by activating STAT3 signaling. In addition, overexpressed CD36 activates PPAR signaling, promotes PPARα and PPARγ expression, and enhances the mitochondrial respiration and cellular oxygen consumption rate (OCR) by cellular lipid metabolism [65]. CD36 can also induce EMT in gastric cancer by mediating the uptake of palmitic acid, leading to the upregulation of DEK and activation of the AKT/GSK-3β/β-catenin signaling pathway, resulting in increased migration and invasion of cancer cells [66]. Moreover, in gastric cancer with peritoneal metastasis (PM), CD36 was shown to promote EMT through PI3K/AKT/mTOR pathway [67]. CD36 has also been shown to exhibit similar biological functions in hepatocellular carcinoma [68], cervical cancer [69], and renal tubular epithelial cells [70]. These findings indicate an important role of CD36 in EMT and suggest that targeting the CD36-mediated EMT pathway can inhibit cancer metastasis.

## 6. CD36 and Tumor Angiogenesis

In parenchymal tumors, angiogenesis is extremely important for tumor proliferation. In 1971, Folkman conjectured that solid tumors larger than 1–2 mm in diameter require neoangiogenesis to sustain their further proliferation [71]. The role of CD36 in regulating tumor microangiogenesis has been confirmed in recent years in multiple ways [72,73], but some recent reports have unraveled new insights into the action of CD36 in regulating tumor vasculature. This suggests the need for further research for in-depth characterization of the role of CD36 in vascular regulation.

### 6.1. CD36 Inhibits Tumor Angiogenesis

TSP-1 is a potent endogenous angiogenesis inhibitor that inhibits angiogenesis by directly affecting the migration, proliferation, survival, and apoptosis of endothelial cells and by antagonizing the activity of VEGF [74,75]. The negatively charged CLESH structural domain residue in CD36 interacts with the TSR to inhibit tumor angiogenesis [76].

For example, in glioma cells, the mechanism of the inhibitory effect of vasopressor (Vstat120) on tumor cell growth and angiogenesis relies on the binding of the TSR sequence therein to the CLESH structural domain in CD36 expressed on microvascular endothelial cells (MVECs) [77]. Klenotic et al. further found that Vstat120 binding to CD36 activated the cystein-mediated pro-apoptotic pathway in MVECs, which in turn inhibited tumor angiogenesis, and that this inhibitory effect could be abrogated by histidine-rich glycoprotein (HRGP) [78]. In breast cancer, CD36 has a similar biological function. On the one hand, in a mouse model, chronic diet-induced obesity was shown to promote vascular remodeling in breast cancer through the LPA/PKD-1-CD36 signaling axis, by significantly reducing CD36 expression, exhibiting its role in inhibiting angiogenesis [79]; on the other hand, CD36 can bind to the N TSP2-Fc fragment to induce caspase-3 production and reduce mitochondrial membrane potential in human dermal microvascular endothelial cells (HDMEC), activating the apoptotic pathway in HDMEC cells, thus acting as an inhibitor of tumor angiogenesis [80]. These findings illustrate the negative regulatory effect of CD36 on tumor vasculature. The inhibitory effect of CD36 on angiogenesis is mainly through binding to TSR in endogenous TSP, promoting apoptosis of endothelial cells and antagonizing the activity of VEGF, which in turn inhibits angiogenesis.

There has been some progress in the development of drugs targeting CD36-TSP-1 [81,82], but no relevant drugs have been marketed yet, and most of the drugs are at the stage of clinical trials. Further research on the mechanism by which CD36 regulates tumor vasculature may enable the development of safe and effective anti-tumor drugs.

### 6.2. CD36 Binds to TSP-1 and TPS-2 to Promote Tumor Metastasis

Similarly, TSP-2, which belongs to the same TSP family of stromal cell proteins as TSP-1, is also a potent inhibitor of tumor angiogenesis [83,84], and its binding to CD36 inhibits tumor angiogenesis. However, in prostate cancer cells, overexpression of TSP-2 was found to promote the expression of matrix metalloproteinase-2 (MMP-2) through the mitogen-activated protein kinase (MAPK) pathway, which in turn promoted tumor cell migration and invasion, and CD36 mediated TSP-2-induced MMP-2 activation and cell migration [85]. In pancreatic ductal adenocarcinoma, TSP-2 activated the MAPK signaling pathway to promote tumor progression [62]. This result seems contradictory to the previous study which showed that TSP-2 can bind to CD36 to inhibit tumor angiogenesis and thus tumor proliferation. In addition, the expression of MMP-2 was shown to be associated with tumor angiogenesis and VM [86,87]. It is suggested that CD36 may bind to TSP-2 to promote tumor angiogenesis through the MAPK signaling pathway. Interestingly, Firlej et al. also suggested that binding of CD36 to TSP-1 promotes prostate cancer cell migration and inhibits angiogenesis; however, the consequent result is increased TME hypoxia, and this hypoxic environment in turn induces TSP-1 to promote tumor cell invasion [88]. These findings suggest that the binding of CD36 to TSP-1 and TPS-2 may have stimulated tumor metastasis and invasion through other pathways, and further research is required to unravel the exact mechanism.

### 6.3. CD36 Mediates VM in Tumors

VM is a newly-defined pattern of tumor perfusion, wherein the tumor cells do not depend on endothelial cells but rather the tumor cells and extracellular matrix aggregate with each other to proliferate rapidly, forming a tubular system with vascular-like structures that anastomose with the host vascular system to form a unique microcirculatory system within the tumor [89,90].

Recently, Martini et al. reported that CD36 could induce VM formation in melanoma cells by promoting the adhesion of tumor cells to the ECM and that this VM formation was not affected by TSP-1 [9]. This finding demonstrated for the first time that CD36 can act as a positive regulator of tumor angiogenesis in contrast to previous results where CD36 inhibited tumor angiogenesis and challenges therapies to counteract tumor angiogenesis via the CD36 pathway. However, there is a paucity of studies exploring the relationship between CD36 and tumor VM and further research is required to investigate the specific mechanisms.

In addition, hypoxia has been reported to increase CD36 expression on MVECs [91], whereas normal angiogenesis and vascular repair depend on CD36 on endothelial cells (ECs) [92]. In addition, CD36 is more abundantly expressed in microvascular ECs than in macrovascular ECs [93]. These results imply that CD36 may promote tumor angiogenesis in some way, which is similar to the findings of Martini et al. [9]; however, the exact mechanism needs to be further investigated. Therefore, CD36-mediated tumor VM may have great research potential in the future, and characterization of the specific underlying mechanism will provide a theoretical basis for the function of CD36 in regulating tumor vasculature.

## 7. Summary and Perspective

CD36 is a multifunctional molecule that plays a key role in cellular lipid absorption, immune regulation, angiogenesis, and tumor proliferation and metastasis. The study of the role of CD36 in various diseases is of far-reaching significance, especially in the context of tumors. In terms of tumor immune regulation, CD36-mediated lipid uptake can induce reprogramming of Treg cells in tumors, promote their lipid metabolism, and exert pro-cancer effects. CD36 can also mediate the uptake of FAs by CD8^+^T lymphocytes, induce intracellular lipid peroxidation and ferroptosis, and weaken the anti-tumor immunity of CD8^+^T lymphocytes. CD36 can also lead to the excessive accumulation of lipids in macrophages, promoting their differentiation to TAMs, and can mediate the fusion of tumor stem cells with TAMs to promote tumor progression. However, there is diversity in the role of CD36 in CAFs. For example, in breast cancer, CD36 is negatively regulated by CAFs and the lower the expression of CD36 in CAFs, the more aggressive the tumor; however, in colon and prostate cancers, high expression of CD36 in CAFs presents the ingested lipids to tumor cells, promoting lipid metabolism in tumors. In addition, CD36 can also mediate EMT in a wide range of tumor cells, promoting tumor progression and metastasis. In terms of tumor angiogenesis, the same versatility of CD36 is demonstrated. On the one hand, CD36 acts as an inhibitor of angiogenesis by binding to TSP-1 and TSP-2; however, it has recently been shown that binding of CD36 to TSP-1 and TSP-2 can promote tumor migration and invasion; on the other hand, CD36 can promote tumor angiogenesis by VM in a manner that is not affected by TSP-1. These findings are somewhat at odds with the previous view that CD36 negatively regulates angiogenesis, and more research is necessary to clarify the specific mechanisms involved.

In summary, the role of CD36 in lipid uptake and vascular regulation affects tumor progression through multiple mechanisms. Targeting CD36 to correct lipid metabolism in TME to mitigate tumor progression is an attractive therapeutic strategy. However, its binding to TSR can, in turn, promote apoptosis of the vascular endothelium and act as an inhibitor of angiogenesis in tumors. Therefore, the functional diversity of CD36 has also influenced the development of relevant clinical agents. Currently, antitumor drugs targeting CD36 are in clinical trials, but their efficacy and safety need to be further improved. Therefore, novel CD36-targeting drugs with low toxicity are a future research trend.

## Abbreviations

FAs	fatty acids
TSP-1	thrombospondin-1
TSP-2	thrombospondin-2
CAFs	cancer-associated fibroblasts
EMT	epithelial mesenchymal transition
VM	vascular mimicry
TME	the tumor microenvironment
OvCa	Ovarian cancer
SR-B2	the class B receptor family
FAT	the fatty acid transporter
LCFA	long chain fatty acids
oxLDL	oxidized low density lipoprotein
oxPLs	oxidized phospholipids
CLESH structural domain	CD36, LIMP-2, Emp sequence homologous
TSR	TSP-1 repeat domain 2
PPAR-β	peroxisome proliferator-activated receptor β
OXPHOS	oxidative phosphorylation
TAMs	tumor-associated macrophages
